# Physical therapists’ assessments, analyses and use of behavior change techniques in initial consultations on musculoskeletal pain: direct observations in primary health care

**DOI:** 10.1186/s12891-016-1173-x

**Published:** 2016-07-27

**Authors:** C. Emilson, P. Åsenlöf, S. Pettersson, S. Bergman, M. Sandborgh, C. Martin, I. Demmelmaier

**Affiliations:** 1 Department of Neuroscience, Uppsala University, Box 5932 S-751 24, Uppsala, Sweden; 2 Division of Physiotherapy, Department of Neurobiology, Care Sciences and Society, Karolinska Institutet, Huddinge, Sweden; 3 Department of Rheumatology, Karolinska University Hospital, Stockholm, Sweden; 4 Research and Development Center Spenshult, Halmstad, Sweden; 5 Department of Public Health, and Community Medicine, Institute of Medicine, The Sahlgrenska Academy, University of Gothenburg, Gothenburg, Sweden; 6 School of Health, Care and Social Welfare, Mälardalen University, Västerås, Sweden

## Abstract

**Background:**

Behavioral medicine (BM) treatment is recommended to be implemented for pain management in physical therapy. Its implementation requires physical therapists (PTs), who are skilled at performing functional behavioral analyses based on physical, psychological and behavioral assessments. The purpose of the current study was to explore and describe PTs’ assessments, analyses and their use of behavioral change techniques (BCTs) in initial consultations with patients who seek primary health care due to musculoskeletal pain.

**Methods:**

A descriptive and explorative research design was applied, using data from video recordings of 12 primary health care PTs. A deductive analysis was performed, based on a specific protocol with definitions of PTs’ assessment of physical and psychological prognostic factors (red and yellow flags, respectively), analysis of the clinical problem, and use of BCTs. An additional inductive analysis was performed to identify and describe the variation in the PTs’ clinical practice.

**Results:**

Red and yellow flags were assessed in a majority of the cases. Analyses were mainly based on biomedical assessments and none of the PTs performed functional behavioral analyses. All of the PTs used BCTs, mainly instruction and information, to facilitate physical activity and improved posture. The four most clinically relevant cases were selected to illustrate the variation in the PTs’ clinical practice. The results are based on 12 experienced primary health care PTs in Sweden, limiting the generalizability to similar populations and settings.

**Conclusion:**

Red and yellow flags were assessed by PTs in the current study, but their interpretation and integration of the findings in analyses and treatment were incomplete, indicating a need of further strategies to implement behavioral medicine in Swedish primary health care physical therapy.

## Background

Behavioral medicine (BM) is based on a bio-psychosocial model of health [1, 2] and is recommended to be implemented in physical therapy practice, e.g., when treating patients with musculoskeletal pain [3–5]. One core feature in BM treatment is the functional behavioral analysis derived from operant learning theory. This analysis aims to describe how key behaviors for goal attainment are determined and maintained by physical, psychological, and contextual factors [2, 6]. It is used as a tool for planning and revising treatment to facilitate long-term behavioral changes, rather than mere symptom reduction, in patients.

Integrating BM into physical therapy requires physical therapists (PTs) who are skilled in assessing and analyzing not only physical but also psychological and behavioral aspects related to a clinical problem. However, PTs have historically been trained in a biomedical tradition focused mainly on analyzing physical symptoms (e.g., pain characteristics); thus, implementing BM in physical therapy has proven to be a great challenge [3, 7–11]. Examples of barriers to implementation are time pressure, perceived lack of skills, and patients’ expectations of biomedical-based treatment [3, 7, 11]. BM education and training have been found to improve PTs’ bio-psychosocial knowledge, attitudes and self-reported skills [12] but it has been pointed out that self-reports of behavior may be biased compared with direct observations [13].

Clinical guidelines for managing musculoskeletal pain recommend first assessing and identifying any signs of severe physical conditions (i.e., “red flags”) and then assessing the psychological prognostic factors of poor outcomes (i.e., “yellow flags”) [14, 15]. Although red flags are frequently assessed by PTs in primary health care, previous research indicate that the assessments must be more specific and detailed [16]. Yellow flags include patients’ negative beliefs and expectations about recovery, anxiety, and fears about pain and injury [17, 18]. These flags play a crucial role in the transition from acute to long-term pain management [17] and are thus important to identify and target at an early stage of a pain condition [19]. According to some reports, yellow flags are not systematically assessed in clinical physical therapy practice [3, 9, 20]. Recordings of brief telephone consultations on musculoskeletal pain in primary health care indicate that PTs can improve their exploration of yellow flags if they are guided step by step and if they receive specific performance feedback [21].

The content in behavior change interventions have traditionally been poorly defined and described in previous studies [22, 23]. To address this problem, a taxonomy of behavior change techniques (BCTs) has been developed [24, 25]. A BCT is an active component of an intervention that is used to support a specific health behavioral change (e.g., increase physical activity, decrease sedentary behavior). Systematic reviews indicate that BCTs targeting self-regulation, e.g., goal setting and self-monitoring of behavior, are the most salient for influencing and maintaining health behaviors [23, 26, 27]. A BCT taxonomy appears useful for describing BM interventions in physical therapy; however, to date, it has been used sparsely in this context.

In summary, no evidence-based behavioral medicine model of clinical reasoning has been fully implemented in physical therapy. Assessing yellow flags and performing functional behavioral analyses in treating patients with musculoskeletal pain appear to be challenging to PTs trained in a biomedical tradition. To inform further implementation, detailed knowledge of the variations in PTs’ clinical practices, indicating which behavioral medicine components are actually used and which are not, is necessary. Video-recorded observations of experienced PTs who are willing to apply a behavioral medicine model of clinical reasoning in their practice may be used to identify areas for improvement and for developing corresponding implementation strategies.

## Method

### Aim

The overall aim of the present study was to explore variation and describe experienced PTs’ observed behaviors when performing initial consultations regarding musculoskeletal pain in primary health care. More specifically, the aims were to investigate PTs’ 1) assessments of red and yellow flags, 2) analysis of clinical problems, and 3) use of BCTs to facilitate pain self-management.

### Design

This study used a descriptive and explorative design with video-based deductive and inductive analyses.

### Participants and data collection

The participants were 12 PTs from six primary care centers in three different counties in Sweden, who signed up to deliver a behavioral medicine intervention targeting musculoskeletal pain within a randomized controlled trial (RCT) [28]. The RCT aimed to study treatment effects on patients and we also conducted a parallel study to describe of PTs’ clinical practice at baseline and their delivery of the intervention. Informed consent was obtained from each participant, who were also informed about how data would be safety stored and how the results would be reported.

Background data and three items on attitudes towards a behavioral medicine model of clinical reasoning [29] and self-efficacy were collected by a study-specific questionnaire before the video recordings (Table 1). Self-efficacy in managing patients with musculoskeletal pain was assessed by two scales: one scale for patients *with* fear of movement and/or catastrophizing (9 items) and one scale for patients *without* fear of movement and/ or catastrophizing (7 items) [30].

**Table 1:** PT background data, attitudes towards a behavioral medicine working model and self-efficacy for managing patients with and without yellow flags (*n* =12)

Background variable	
Sex, female *n*	12
Age, mean (range)	50 (39-57)
Years in the profession, mean (range)	19 (10-35)
Years in primary healthcare, mean (range)	14 (3-28)
Further education	
Behavioral medicine or cognitive behavioral therapy, *n*	6
Motivational interviewing, *n*	5
Pain treatment/rehabilitation, acupuncture, *n*	7
Orthopedic manual therapy, *n*	8
Other courses^a^ *, n*	12
Attitudes towards a behavioral medicine working model for clinical reasoning Md *(IQR)*	
1. How important is it for you to work according to a behavioral medicine model for clinical reasoning? (NRS 0-10)^b^	8.5 (2.5)
2. How confident are you to work according to a behavioral medicine model of clinical reasoning? (NRS 0-10)^b^	6.0 (2.7)
3. How ready are you to work according to a behavioral medicine model of clinical reasoning? (NRS 0-10)^b^	7.0 (4.5)
Self-efficacy Md *(IQR)*	
Managing patients with fear of movement and/ or catastrophizing^c^ (0-90)	60.0 (45.0)
Managing patients without fear of movement and/ or catastrophizing^d^ (0-70)	45.0 (28.0)

^a^McKenzie method of mechanical diagnosis and therapy (MDT), medical exercise therapy, specific manual treatment of the joints, body awareness treatment, sports medicine, educational courses and physical activity and exercise. ^b^ rating scale 0-10, where 0 = not important at all/ low confidence/ not ready at all, and 10 = extremely important/ high confidence/ completely ready ^c^ 9 items, rating scale 0-10, where 0 = low self-efficacy and 10 = high self-efficacy

^d^ 7 items, rating scale 0-10, where 0 = low self-efficacy and 10 = high self-efficacy

Data on the PTs’ clinical behaviors were collected by video recordings during initial consultations with patients seeking care for musculoskeletal pain; the patients were scheduled according to the usual routines. Additional data on clinical behaviors were collected by interviews.

The video recordings were performed by one of the authors (SP) at the primary care center where the participants were working. The video camera was placed in a fixed position using a single viewpoint with focus on the PT during the consultation, and the researcher was an observer. Before the video recordings began, the researcher ensured that patients were comfortable with the camera’s presence and oral informed consent was obtained from every patient. The video recordings lasted 19–69 min (mean 39 min) and took place between June 2013 and January 2014.

To assess whether the PTs performed covert observations and analyses that were not captured in the video recordings, the third author (SP) interviewed each PT, either immediately after the consultations or during the next day. Three or four video sequences from each consultation representing main parts of assessment, analysis and treatment were used for stimulated recall by interview. The interview guide used open-ended questions as follows: “What were your thoughts in this situation?”, “How did you reason in in this situation?”, “What was your intention with your question?” and “What do you think when you watch this video sequence?” used as appropriate depending on the recorded situation. The PT was encouraged to stop the video if she wanted to comment on something specifically. The interviews lasted 12–35 min (mean 22 min).

### Data management and analyses

The descriptive variables of the PT’s were calculated using means (range), and medians (interquartile range) as appropriate. The video recordings were analyzed focusing on three domains (i.e. PTs’ assessment of red and yellow flags, their models of analyses, and their use of BCTs) using a combined deductive and inductive approach. All recordings were first reviewed by CE and ID separately to get an overview of the content. *A deductive analysis* (see below for details) was performed by CE based on a protocol with pre-defined criteria (Table 2), and a taxonomy of BCTs [25]. Relevant video-sequences for the three domains in all 12 video recordings were identified and transcribed by CE. The transcriptions were analyzed and organized by CE and ID, including a refined coding of specific BCTs, resulting in a summarizing description of all 12 cases (Table 3). *An inductive analysis* (see below for details) was then performed to describe the variation in the PTs’ clinical practice. The video sequences were again reviewed several times by CE and illustrative cases were identified in discussions with ID, CM and PÅ. The interview data were analyzed separately by CE, ID and SP, and discussed until consensus was reached.

**Table 2:** Observation protocol for PTs’ assessment of red and yellow flags

Definition of red flags	Examples of PTs’ questions to assess red flags
Trauma	Have you experienced any trauma related to your pain?
Severe diagnosis	Have you had cancer or any other severe diagnosis?
Severe spinal pathology	Have you experienced radiating leg pain?
Patterns or symptoms not related to mechanical pain	How is your health in general?
Numbness and paresthesia in the perianal region	Have you had any bowel and bladder disorders?
Difficulty with micturition	
Weakness or numbness in the legs related to back pain	Have you felt any weakness or numbness in your legs?
Definition of yellow flags	Examples of PTs’ questions to assess yellow flags
Beliefs, appraisal, and judgments	
Unhelpful beliefs about pain	What are your thoughts about the pain?
Expectations of poor treatment outcome	What are your thoughts about recovery?
	What do you think about your capability to work?
	Which factors do you think affect your pain?
Emotional responses	
Distress not meeting the criteria for diagnosis of mental disorder	Are you worried about your pain?
	How does the pain affect your mood?
Worry, fears and anxiety	Are you distressed about the pain?
	Are you avoiding doing any activities due to pain?
Pain behavior and coping strategies	
Avoidance of activities due to expectations of pain and possible re-injury	Do you avoid activities due to pain?
	What are you doing when having pain?
Over-reliance on passive treatments	What are your thoughts about pain relief?
	What activities are difficult for you due to pain?

**Table 3:** Summary of behavior change techniques (BCTs) that were used in the 12 consultations, organized in specific groups and codes according to the BCT taxonomy (v.1) (Michie et.al. 2013)

Used BCT’s	Frequency of BCTs (=*n)*	Examples
*1. Goals and planning*		
1.1 Goal setting (behavior)	1	The PT guided the patient in setting a goal for regular walking or bicycling in order to improve overall health.
1.3 Goal setting (outcome)	1	The PT guided the patient in setting a goal in terms of weight loss.
*2. Feedback and monitoring*		
2.2 Feedback on behavior	2	The PT made suggestions to the patient, who had performed her aqua exercise at too high intensity.
*4. Shaping knowledge*		
4.1 Instruction on how to perform the behavior	9	The PT instructed the patient in a home-based exercise program.
*5. Natural consequences*		
5.1 Information about health consequences	12	The PT informed the patient about the positive consequences of muscle strength exercise in osteoarthritis.
*7. Associations*		
7.1 Prompts/cues	1	The PT discussed with the patient how to use a daily activity as a reminder of the home-exercises she was to perform
*8. Repetition and substitution*		
8.1 Behavioral practice/rehearsal	1	The PT asked the patient to repeat and demonstrate the exercises she had been instructed to do in order to remember them better.

#### The deductive analysis

An observation protocol was developed to categorize the PTs’ clinical behaviors:

1. Assessment of red flags. To be judged as having assessed red flags, the PTs had to ask at least one question specifically about severe symptoms and other severe diagnoses, traumas or spinal pathology in combination with a question about general health (Table 2).
2. Assessment of yellow flags. To be judged as having assessed yellow flags, the physical therapists had to ask at least one question about specific psychological prognostic factors [18] according to the protocol (Table 2).
3. Analysis of the clinical problem. To be judged as having performed any analysis at all, the PT had to assess and evaluate findings by breaking down the clinical problem into parts and discuss with the patient a hypothetical cause-effect relationship between those findings. The analysis was categorized as one of three models: a) functional behavioral, b) bio-psychosocial, or c) biomedical. For an analysis to be categorized as functional behavioral, the pain problem had to be defined in behavioral terms, and functional relationships between individual and contextual factors had to be identified and discussed with the patient (e.g. how a patient’s fear of increased pain results in avoidance behaviors that are negatively reinforced by subsequent reductions in fear, which in turn may impedes participation in important activities) [2, 6]. For an analysis to be categorized as bio-psychosocial, both biomedical and psychosocial factors had to be integrated, but not related to the behavioral learning mechanisms as described above. A biomedical analysis had to be based solely on physical assessments, physical impairments or limitations and the physical environment (i.e., high curbs or stairs).

Assessment of BCTs was measured using the BCT taxonomy [25]. The hierarchically organized taxonomy comprises 93 well-defined and consensually agreed-upon BCTs, i.e. observable, replicable and irreducible intervention components intended to facilitate behavior change, grouped into 16 clusters [25]. The taxonomy is organized with labels, definitions and clinical examples of each BCT. The overall inter-coder reliability of the BCT taxonomy is good [31].

#### The inductive analysis

Variation among PTs was explored by a) identifying different combinations of their ways to assess, analyze and use BCTs and, b) identifying their different ways to interact with their patients during the consultations, e.g. more or less patient-centered in terms of asking questions, listening and involving the patient.

Four of the 12 cases deemed to illustrate different and clinically relevant variations were selected. The four cases are presented with brief descriptions of each PT’s background, narratives and quotations. Pauses, hesitating words, sounds, and quickening or slowing of the speed in conversations were omitted in the transcripts, and ellipses (…) indicate omitted material in the quotations.

CE, ID, MS and PÅ were PTs with clinical experience treating patients with musculoskeletal pain and with expertise in integrating behavioral medicine in physical therapy. One of the authors (SP) was a nurse with experience of treating patients with pain conditions and with expertise in qualitative research. CM was a PT with expertise in qualitative research methods, including video-based analysis. SB was a general practitioner and pain physician.

### Ethical considerations

The potential harm for the participants might be negative experiences from being video-recorded and observed. It may also challenge their professional confidence and beliefs regarding their role as physiotherapists. For the patients, a potential risk of threats to privacy and confidentiality during the video-recordings could have occurred, and they may have felt uncomfortable in the situation. However, the quotations and personal information about the patients are only briefly described and not possible to relate to a specific patient. The patients were informed that the video-recordings would be stopped if they wished, and that focus in the observation was on the PTs, not on the patients.

## Results

### Description of participants

Participants’ characteristics are presented in terms of background data, attitudes and self-efficacy (Table 1). All PTs were women, well experienced and six of them had some formal training in working according to BM principles.

### Assessment of red and yellow flags

Red flags were assessed in nine out of 12 consultations. There were no positive findings of red flags in any of the cases (Table 4).

**Table 4:** Patient pain-sites, PTs’ assessment and identification of red and yellow flags in 12 video recordings

	Pain *s*ite(s)	Assessment of red flags	Identification of red flags	Assessment of yellow flags	Identification of yellow flags
	*n*	*n*	*n*	*n*	*n*
Low back	3	4	0	2	0
Shoulder	2	3	0	2	2
Hip	4	1	0	3	1
Head	1	1	0	1	1
Low back and foot	1	0	0	0	0
Shoulder and elbow	1	0	0	0	0
Total	12	9	0	8	4

Yellow flags were assessed in eight out of 12 consultations, with positive findings in four of them (Table 4). The identified yellow flags represented pain behaviors, coping strategies and emotional responses (Table 5).

**Table 5:** Description of the video observations based on the assessments of red and yellow flags, analyses and BCTs. Cases 1-4 were selected to illustrate the clinical variation

PT	Red flags		Yellow flags		Pain site	Functional behavioral analysis	Biopsycho- social analysis	Biomedical analysis	BCTs^a^	Time for consultation (minutes)
	Assessment	Identification	Assessment	Identification						
1	Red flags were assessed but not identified	No	Yes	Passive coping strategies	Headache	No	Yes, the analysis included yellow flags in relation to symptoms/pain problem but not to a specific behavior.	Included in the functional behavioral analysis	*1.1: Behavior goal setting* Regular physical activity (walking) *4.1: Instruction on to perform the behavior* Home exercise for headache *5.1: Info about health consequences* Physical exercise with headache, how muscle tension leads to headache	24
2	Red flags were assessed but not identified	No	Yes	Negative thoughts, avoiding behavior related to physical activity and the shoulder	Shoulder	No	No	Yes, short analyses during the consultation with focus on the shoulder pain. Yellow flags were not included in the analysis	*4.1: Instruction on how to perform the behavior* Exercises for the shoulder *5.1: Info about health consequences* Effect on pain and physical function of the exercises	51
3	Red flags were assessed but not identified	No	No	No	Hip joint	No	No	Yes, short analyses during the consultation	*2.2: Feedback on behavior* Performance of a specific exercise for the hip, walking pattern *4.1: Instruction on how to perform the behavior* Exercise for the hip, circulation exercises, walking with crutches *5.1: Info about health consequences* How exercise affects the hip, pain and function *7.1: Prompts/cues* Connect the exercise with another daily activity *8.1: Behavioral practice/rehearsal* PT asked the patient to repeat the exercises she had instructed her in so that she would better remember them.	25
4	Red flags were assessed but not identified	No	No	No	Shoulder and elbow	No	No	No analysis related to the patient’s problem was performed, but there was a general explanation of the physical findings	*4.1: Instruction on to perform the behavior* Stretching of elbows, home exercises, posture, micro pauses *5.1: Info about health consequences* Factors that increase pain in the shoulder and elbow, how specific work tasks can affect shoulder pain and function	61
5	Red flags were assessed but not identified	No	Yes	No	Low back	No	No	Yes, a summarizing analysis related to the patients pain problem. Commentaries about the assessments and findings during the consultation.	*1.1: Outcome goal setting* Lose weight to decrease back pain *4.1: Instruction on to perform the behavior* Exercise for home-training *5.1: Info about health consequences* How physical activities may affect back pain, pain coping strategies, including exercises	28
6	No	No	Yes	Thoughts and worries regarding other possible causes of pain.	Hip joints	No	No	Yes, short analysis based on biomedical examination and the interview with the patient.	*2.2:Feedback on behavior* Discussion with the patient about how to adjust the intensity of the aquatic exercise following overuse. *2.7: Feedback on outcome* Feedback on improving hip flexibility compared with earlier physical function and exercise *4.1:Instruction on to perform the behavior* Exercise for lower extremities *5.1: Info about health consequences* Consequences for hip and back of wearing high heels	39
7	Yes	No	No	No	Low back	No	No	Yes, analyses including online commentaries and a summary analysis.	*5.1: Info about health consequences* Factors that causes back pain and what the patient can do by herself to manage the pain	25
8	Yes	No	Yes	No	Low back and leg	No	No	Yes, short analyses during the examination and a summary analysis at the end.	*4.1: Instruction on to perform the behavior* Stretching exercises and posture *5.1: Info about health consequences* How running can affect back pain and general health how bad body position/posture behavior can affect back-pain	19
9	No	No	Yes	No	Hip joints	No	No	Yes, short analysis of hip pain during the examination and a summary analysis	*4.1: Instruction on to perform the behavior* Walking on stairs, exercises for hips *5.1: Info about health consequences* Effects of exercise and bicycling in osteoarthritis	69
10	Yes	No	Yes	Avoiding dancing and fear of increased pain in the shoulder	Shoulder	No	No	Yes, short analysis of shoulder pain during the examination and a summary analysis	*4.1: Instruction on how to perform the behavior* Exercise for shoulders *5.1: Info about health consequences* How heavy lifting and other work tasks affect shoulder pain	47
11	No	No	No	No	Feet and low back	No	No	Yes, analysis related to the patient’s foot pain	*5.1: Info about health consequences* How exercise and body weight affect foot problems, changes in foot status due to ageing and injuries have consequences in pain and medicine consumption *15.1: Verbal persuasion about capability* Benefits of physical exercise, patients capabilities for exercise	39
12	Yes	No	Yes	No	Hip joints	No	No	Yes, a summary analysis at the end and a short online commentaries during the consultation	*2.2: Feedback on behavior* How to do yoga exercises *4.1: Instruction on how to perform the behavior* Stretching of hip muscles, home exercises *5.1: Info about health consequences* How physical exercise and activity affect the cartilage in the hip *7.1: Prompts/cues* How to use daily activities as a cue to action (home exercises)	36

*PT* physical therapist. ^a^ *BCT* behavior change technique presented in specific codes

### Model for analysis

Biomedical analyses were performed in ten out of 12 consultations, and a bio-psychosocial analysis was performed in one consultation. None of the analyses met the criteria for a functional behavioral analysis, and in one case, no analysis at all was performed (Table 5).

The interviews with the PTs, performed to assess any covert analyses related to the selected video sequences supported the interpretation of the results from the video recordings.

### Use of behavior change techniques

BCTs were used by all PTs to facilitate physical activity, e.g. improve posture. The most frequently used BCTs concerned informing or instructing the patient, such as providing information about health consequences. BCTs concerning goals and planning were observed in two of the 12 consultations. Examples of BCTs used by the PTs are described in Table 3.

### Variations in clinical practice

An overview of all 12 cases are presented in Table 5. Variations in the PTs’ clinical practice were found regarding the three domains, but also in the ways they communicated with their patients. The four selected cases illustrated various degrees of patient involvement. One of the PT’s was involving the patient in communication to a great extent in combination with a bio-psychosocial analysis of the clinical problem (Case 1). Another PT involved the patient in the communication regarding the patients’ history and assessment of yellow flags, but did not integrate a bio-psychosocial model in her analysis or treatment (Case 2). Two of the PTs used closed questions and were more focused on the patients’ physical symptoms in various degree; one was partly involving the patient in the treatment discussion (Case 3), while one PT demonstrated a more traditional approach not considerate the patient’s participation in discussions and decisions (Case 4). Detailed descriptions of the selected cases are presented below.

#### Case 1: High degree of patient involvement and a biopsychosocial analysis

This case was selected to demonstrate a PT who performed a bio-psychosocial analysis and guided a patient in behavioral goal setting. The PT had many years of experience in the profession and had taken a number of courses in manual treatment and in motivational interviewing (MI). The patient was a woman seeking care for headache. The PT assessed red flags with no positive findings. Yellow flags were assessed, identifying passive pain coping strategies and worries related to a stressful family situation. Analysis of the clinical problem was based both on the physical examination and the identified yellow flags. The PT performed a summarizing bio-psychosocial analysis at the end of the consultation with the patient, but explained the assessment and findings during the examination. The patient was involved in sharing information, decision-making, power and responsibility regarding the assessment, analysis and treatment. The BCTs used by the PT to promote home-based exercise were behavioral goal setting, instruction on how to perform the behavior, and information about health consequences (i.e., effects of physical exercise on headaches and the relationship between muscle tension and headache).

Quotes: Bio-psychosocial analysis

- *PT: “ That’s the way it works with tension headache…tension is stored in the muscles and tightens up without you noticing it…then there won’t be any clearing of waste products in the muscles; rather, waste stays and causes pain instead. But then there is also the other thing you talked about…the situation in the family that is also affecting the pain…”*
- *Patient: “ Yes, that’s correct… and maybe it is good then to be able to relax a little?”*
- *PT: “ Absolutely, but it may not work to start with all the physical activity at once… But something you can do…try to recognize what is happening in the body when it is tense. It seems to be in the evening that it is triggered, is that correct?”*

Quotes: BCT Behavioral goal setting

- *PT: “Are you usually physically active?”*
- *Patient: ” Not as much I would like”*
- *PT: “How much would you be able to do?”*
- *Patient: “To be able to take a walk around the area where I live…maybe 20 minutes”*
- *PT: “Is that a goal you would like to work for?”*
- *Patient: “Yes, it is absolutely a goal”*
- *PT: “How would you be able to do that? Can you find situations where that would work?”*
- *Patient: “I suppose it is just to do it…Just leave everything at home…maybe my son could cycle beside me…if I cannot leave him alone”*
- *PT: “It might be a good strategy”*

#### Case 2: Some patient involvement and a biomedical analysis

This case was selected to demonstrate the assessment and identification of yellow flags that were not integrated in the analysis or related to the BCTs used by the PT. The PT had many years of experience in the profession, further education in physical activity including a basic course in MI. The patient was a female who was seeking care for shoulder pain. Red flags were assessed with no positive findings. The following yellow flags were identified: negative thoughts about recovery and treatment outcome, avoiding behavior related to physical activity in general and avoiding use of the shoulder in daily activities. In the initial interview, the PT used open questions, considered the patients’ experiences, thoughts and feelings from a bio-psychosocial perspective. She performed a physical examination related to the clinical symptoms and a short analysis during the examination, based only on the physical findings. The BCTs used by the PT to promote home-based exercise were instruction on how to perform the behavior and information about health consequences (i.e., effects of physical exercise on shoulder pain and the importance of being physically active).

Quotes: Assessment and identification of the following yellow flags:

- Pain behavior and coping strategies
  *PT: “Is there anything you can do that would improve your shoulder problem?”*
  *Patient: “No, I don’t think so… I have tried everything”*
  *PT: “What have you tried?”*
  *Patient: “I have had my arm tied on to my body when I walk around at home…because when hanging it down, it starts to ache. I keep it still and use the right arm instead.”*
  *PT: “Do you use the left arm at all?”*
  *Patient: “No, it’s just there…I’d rather just cut it off”*
- Beliefs, appraisal, and judgments/emotional responses
  *PT: “Did you meet any rehab personnel at the hospital?”*
  *Patient: “No, I came to the trauma section…with the ambulance…and then I was discharged after 3 days, and after that, nothing much happened…it is really as if no one ever listened to my problems…”*
  *PT: “It sounds as if you are rather disappointed”*
  *Patient: “Yes, I am quite bitter about it really… but now so much time has gone by that nothing can be done, but I am worried that it may happen again”*

#### Case 3: Minor patient involvement and a biomedical analysis

This case was selected to demonstrate how a few BCTs can be used in a pedagogical and structured way. The PT had many years of experience in the profession and had taken courses in manual treatment of pain. The patient was a woman who needed rehabilitation after hip replacement surgery. No assessment of red or yellow flags was performed. The PT performed a physical examination of the hip and lower extremities, and the analysis was based solely on biomedical findings. A number of BCTs were used to promote home-based exercise: feedback on behavior, instruction on how to perform the behavior, information about health consequences, prompts and cues and behavioral practice with rehearsal. The communication was mainly focused on biomedical factors and directed by the PT, but the patient was involved in the discussion about the home-based exercises.

Quotes: BCT Prompts and cues

- *PT: “When would it work to do the exercises?…Is there any particular instance in relation to something else that you are doing and that recurs during the day?”*
- Patient: *“It could work if I do it the same time as when I get ready in the evening…”*

Quotes: BCT: Behavioral practice/rehearsal The patient repeated the exercises together with the PT until the PT was sure that the patient could manage them on her own.

- *PT: ”If we summarize the exercises we have just gone through, can you repeat what we have done and see what you can remember? What did we do now? What will you think about? Can you repeat what we have done and tell me what you should remember about it?”*

#### Case 4: Very low degree of patient involvement and no analysis

This case was selected to demonstrate a traditional biomedical model of clinical reasoning in which the PT was active and the patient played a passive role. The communication was strictly directed by the PT and consisted mainly of closed questions. The PT had many years of experience in the profession and had taken multiple courses in manual treatment and a course in cognitive behavioral therapy for PTs. The patient was a man who was seeking care for shoulder and elbow pain. Red flags were assessed, with no positive findings; yellow flags were not assessed. A general biomedical hypothesis regarding the relationship between pain and work was formulated by the PT, but it was not specifically based on the patient’s problem or assessments based on the observation protocol. The main part of the consultation consisted of the PT providing the patient with information. The BCTs used by the PT to promote home-based exercises, enhance body posture and improve working technique were as follows: instruction on how to perform the behavior (exercises) and information about health consequences (physical activity, posture and work-related factors).

Quotes: BCT Information about health consequences. The PT was explaining the consequences of overloading the shoulder in maladaptive positions to the patient.

- PT: “*I have good exercises that I can give you for both the shoulder and the elbow…but it is like this with us humans that the more we can prevent development of pain, the easier it is to feel good and be able to feel good for a long time. By only fixing the pain you will not get better in the long run.”*
- Patient: “*Hmm”*
- PT: “*It is like having a blister and just putting on a plaster and avoiding using the shoes that causing it. It is the same principle, isn’t it?”*
- Patient: “*Hmm”*
- PT: *“Imagine that overloading the shoulder is like having a blister…, you have to think in the same way. Then, I know of course…that in both your and my jobs, it is not possible to take away all the activities that are too strenuous. If you do, you may not have a job to go to after that (laugh).”*

## Discussion

The current study adds new knowledge regarding experienced PTs’ observed clinical behaviors, providing detailed information from initial consultations regarding musculoskeletal pain in primary health care. Observable variation was found concerning the PTs’ assessment of red and yellow flags, the models they used for analysis, their use of BCTs to facilitate pain management, and communication style.

Red flags were frequently assessed in the present study, especially in cases of back and shoulder pain, in line with the clinical guidelines for pain management [32–34].

A positive finding was that a majority of the PTs assessed yellow flags, indicating awareness of the importance of psychological risk factors. However, when yellow flags were found, few of the PTs assessed them any further, and only one integrated them into the analysis of the patient’s clinical problem. These results are in line with previous studies, reporting that PTs acknowledge the importance of yellow flags [20] and that they feel capable of assessing yellow flags but also find it difficult to integrate those assessments in clinical practice [9], while Singla et.al., found that PTs had limited understanding and poor awareness about yellow flags [8].

Nearly all of the PTs performed biomedical analyses of the clinical problem during the consultations. These results are concordant with those of earlier studies on PTs demonstrating biomedical preferences and difficulties in integrating psychosocial factors in assessments, analysis and treatment of musculoskeletal pain [11], confirming that the biomedical tradition in physiotherapy is still dominating.

BCTs were used by all PTs, primarily to promote physical activity behaviors such as home-based exercise; in a few cases, they were associated with a specific behavioral or outcome goal. The most commonly used BCTs were informing patients about health consequences and instructing them on how to perform behaviors, while techniques associated with self-regulation (e.g., goal setting) were rare. Similar findings have been reported in previous studies [35, 36], but others report PTs as using goal setting in clinical practice [37, 38]. Not surprisingly, PTs choose techniques that they feel competent and comfortable to use [9] and the most frequently used BCTs in the present study are the ones used by tradition in PT and healthcare in general.

Several barriers to changing clinical practice towards application of BM have been reported previously: patients’ biomedical treatment expectations, PTs long biomedical tradition, and financial incentives in the health care systems putting time pressure on clinics and health professionals [3, 11, 37, 39]. Time is often mentioned as an implementation barrier in healthcare, as it is assumed it takes more time to perform new clinical behaviors than staying with old routines. However, one case in the present study demonstrated how a bio-psychosocial analysis was performed in half the time it took to perform a consultation with no analysis at all, although both PTs were experienced and well-educated. It is plausible that circumstances other than time pressure are more important, such as patient characteristics and PTs’ attitudes and beliefs about their own behavioral capacity. Lack of confidence and uncertainty to manage psychosocial factors among PTs are other hindering factors to consider [11].

A number of the PTs in the current study were educated in BM but no one performed functional behavioral analyses. Such analyses focus not only on pain history, but on the consequences of pain, how pain-related behaviors are maintained and how they can be changed to improve activity and health [4, 40]. These important links must be understood and explained in collaboration between PTs and patients to provide effective strategies for pain management. Developing skills to do that may not come automatically for PTs by taking a course, but needs time, practice and feedback to actually be implemented in clinical practice [3, 7, 12]. Previous research has found that the extent of education and skills training may be of importance [20]. Eight days of psychosocial assessment and management training had an impact on PTs’ attitudes, beliefs and behaviors [12], and one and a half days of training in basic cognitive behavioral principles improved the assessment of yellow flags [35]. However, very little improvement in psychosocial assessment was seen after a five-hour educational session that included a bio-psychosocial model of clinical reasoning [41]. These findings indicate that PT training in this field should consider not only the content but also the format and time frames of the education. A few PT studies have reported promising results of adherence to a specific bio-psychosocial treatment protocol with systematic training and supervision [38, 42]. on skills training in a step by step format with support and feedback on behavior [43] Video-recording can also be a useful tool in clinical skills training, providing good opportunities for feedback [44].

The variation in how the PTs performed the consultations reflected an association between the bio-psychosocial model of analysis and a patient-centered communication involving the patients’ preferences for information and exercise (Case 1). Patient- centered communication emphasizes the patient’s perspective with open-ended questions integrating the patient’s thoughts and experiences [45, 46] and corresponds well with a biopsychosocial and BM approach. In contrast, a traditional practitioner-centered communication style uses closed questions directed by the health professional, corresponding with a biomedical reductionist approach focusing on diagnosis and biological processes. When a biomedical model was applied the communication was less patient-centered and more directed by the PT (Case 3 and 4). In one case the PT used a patient-centered communication in the interview and assessment, but left out the bio-psychosocial findings and switched over to a more biomedical focused approach (e.g. pain symptoms) when it came to analysis and treatment (Case 2). One explanation for this may be that she had theoretical knowledge, but not the necessary skills to apply a biopsychosocial model throughout the assessment, analysis and treatment. Further education in patient-centered communication for PTs has been suggested in a previous study [47], which is in line with a bio-psychosocial model for clinical reasoning. However, there is no consensus whether the patient-centered communication improve outcomes [48].

### Strengths and limitations

The focus in the current study was to describe the PTs’ clinical practice, not the patients’ perspectives. One strength was that all eligible PTs agreed to participate in the study, possibly due to the fact that they were all highly motivated to implement behavioral medicine and that they had signed up to deliver an intervention within an RCT in primary health care. Thus, they cannot be considered representative of all PTs working in primary health care but rather represent motivated, interested and well-experienced PTs working in primary health care settings. One limitation was our brief information about the PTs prior knowledge of the patients’ health status, which could have influenced the interviews and assessments during the consultation. However, all were initial consultations in the sense that they were not part of an ongoing treatment series.

We combined deductive and inductive analyses, using quantitative and qualitative data. This methodological choice was made to provide a focused but still illustrative picture of PTs clinical practice and may thus be considered as a strength. On the other hand, it limits the use of the existing, rich data from the video recordings, as we for readability reasons had to balance such data against the quantitative data. However, the research questions guided us in the methodological decisions and the analytic procedure is thoroughly described.

The data collection method in this study was video-based observation, which has advantages and limitations [13]. Video observation provides audio and visual details of situations, interactions and specific behaviors that cannot be captured in interviews and surveys [44]. However, video observation does not allow for further investigation of a specific situation: you get what you see. To complement our video data, we conducted additional interviews, which validated the content of the video recordings. It is important to consider practical implications for the participants such acceptability and risks in video-based research [49]. Our use of video recordings may have influenced the PTs’ and patients’ behaviors in the actual situation. However, this has been reported to occur only minimally [44, 50]. A fixed camera position, as used in this study, has been described as being less demanding than a roving camera on participants [13].

The use of a BCT taxonomy, including specific definitions and examples of BCTs, was useful for coding PT behavior, and it added a deductive component to the inductive analyses. If used in a coherent way, the taxonomy enables the accumulation of knowledge as well as future comparisons with other studies of behavioral change in patients and providers.

## Conclusions

The results in this study indicate variation in experienced PTs’ assessments, analyses and of communication during initial consultations with patients presenting with musculoskeletal pain. They also indicate a need of further efforts to implement BM in physical therapy. The assessment of psychological prognostic factors may not be the greatest challenge to PTs; instead, the greatest challenge might be the interpretation and integration of such findings in functional behavioral analyses, goal setting and treatment plans. Future initiatives may use more extensive interviews combined with video observations to explore and to subsequently facilitate changes in PTs’ clinical reasoning.

## Abbreviations

BCT, behavior change technique; BM, behavioral medicine; PT, physical therapist

## References

[1] Fordyce W (1976). Behavioral methods for chronic pain and illness.

[2] Bandura A (1986). Social foundations of thought and action: a social cognitive theory.

[3] Foster NE, Delitto A (2011). Embedding psychosocial perspectives within clinical management of low back pain: integration of psychosocially informed management principles into physical therapist practice--challenges and opportunities. Phys Ther.

[4] Main CJ, George SZ (2011). Psychologically informed practice for management of low back pain: future directions in practice and research. Phys Ther.

[5] Foster NE, Hill JC, Hay EM (2011). Subgrouping patients with low back pain in primary care: Are we getting any better at it?. Man Ther.

[6] Haynes SN, Leisen MB, Blaine DD (1997). Design of individualized behavioral treatment programs using functional analytic clinical case models. Psychol Assess.

[7] Nielsen M, Keefe FJ, Bennell K, Jull GA (2014). Physical therapist-delivered cognitive-behavioral therapy: a qualitative study of physical therapists’ perceptions and experiences. Phys Ther.

[8] Singla M, Jones M, Edwards I, Kumar S (2015). Physiotherapists’ assessment of patients’ psychosocial status: are we standing on thin ice? A qualitative descriptive study. Man Ther.

[9] Sanders T, Foster NE, Bishop A, Ong BN (2013). Biopsychosocial care and the physiotherapy encounter: physiotherapists’ accounts of back pain consultations. BMC Musculoskelet Disord.

[10] Sandborgh M, Åsenlöf P, Lindberg P, Denison E (2010). Implementing behavioural medicine in physiotherapy treatment. Part II: Adherence to treatment protocol. Adv Physiother.

[11] Synnott A, O’Keeffe M, Bunzli S, Dankaerts W, O’Sullivan P, O’Sullivan K (2015). Physiotherapists may stigmatise or feel unprepared to treat people with low back pain and psychosocial factors that influence recovery: a systematic review. J Physiother.

[12] Overmeer T, Boersma K, Main CJ, Linton SJ (2009). Do physical therapists change their beliefs, attitudes, knowledge, skills and behaviour after a biopsychosocially orientated university course?. J Eval Clin Pract.

[13] Heath C, Hindmarsh J, Luff P (2010). Video in qualitative research: analysing social interaction in everyday life.

[14] Koes BW, van Tulder M, Lin CW, Macedo LG, McAuley J, Maher C (2010). An updated overview of clinical guidelines for the management of non-specific low back pain in primary care. Eur Spine J.

[15] van Tulder M, Becker A, Bekkering T, Breen A, del Real MT, Hutchinson A (2006). European guidelines for the management of acute nonspecific low back pain in primary care. Eur Spine J.

[16] Ferguson FC, Morison S, Ryan CG (2015). Physiotherapists’ understanding of red flags for back pain. Musculoskeletal Care.

[17] Linton SJ, Shaw WS (2011). Impact of psychological factors in the experience of pain. Phys Ther.

[18] Nicholas MK, Linton SJ, Watson PJ, Main CJ (2011). “Decade of the Flags” working group. Early identification and management of psychological risk factors (“yellow flags”) in patients with low back pain: a reappraisal. Phys Ther.

[19] Boersma K, Linton SJ (2005). Screening to identify patients at risk: profiles of psychological risk factors for early intervention. Clin J Pain.

[20] Gray H, Howe T (2013). Physiotherapists’ assessment and management of psychosocial factors (Yellow and blue flags) in individuals with back pain. Phys Ther Rev.

[21] Demmelmaier I, Denison E, Lindberg P, Asenlof P (2012). Tailored skills training for practitioners to enhance assessment of prognostic factors for persistent and disabling back pain: four quasi-experimental single-subject studies. Physiother Theory Pract.

[22] Michie S, Fixsen D, Grimshaw JM, Eccles MP (2009). Specifying and reporting complex behaviour change interventions: the need for a scientific method. Implement Sci.

[23] van Achterberg T, Huisman-de Waal GG, Ketelaar NA, Oostendorp RA, Jacobs JE, Wollersheim HC (2011). How to promote healthy behaviours in patients? An overview of evidence for behaviour change techniques. Health Promot Int.

[24] Michie S, Johnston M, Francis J, Hardeman W, Eccles M (2008). From theory to intervention: mapping theoretically derived behavioural determinants to behaviour change techniques. Appl Psychol.

[25] Michie S, Richardson M, Johnston M, Abraham C, Francis J, Hardeman W (2013). The behavior change technique taxonomy (v1) of 93 hierarchically clustered techniques: building an international consensus for the reporting of behavior change interventions. Ann Behav Med.

[26] Fjeldsoe B, Neuhaus M, Winkler E, Eakin E (2011). Systematic review of maintenance of behavior change following physical activity and dietary interventions. Health Psychol.

[27] Greaves CJ, Sheppard KE, Abraham C, Hardeman W, Roden M, Evans PH (2011). Systematic review of reviews of intervention components associated with increased effectiveness in dietary and physical activity interventions. BMC Public Health.

[28] Åsenlof P, Demmelmaier I, Emilson C, Pettersson S, Bergman S (2013). STEP-UP: an innovative stepped-care protocol for tailored behavioral medicine treatment in the management of musculoskeletal pain in primary care. Ann Rheum Dis.

[29] Miller WR, Rollnick S (2002). Motivational interviewing: preparing people for change.

[30] Bandura A, Pajares F, Urdan T (2006). Guide to the construction of self-efficacy scales. Self-efficacy beliefs of adolecents.

[31] Abraham C, Wood CE, Johnston M, Francis J, Hardeman W, Richardson M (2015). Reliability of identification of behavior change techniques in intervention descriptions. Ann Behav Med.

[32] Sizer PS, Brismee JM, Cook C (2007). Medical screening for red flags in the diagnosis and management of musculoskeletal spine pain. Pain Pract.

[33] Henschke N, Maher CG, Ostelo RW, de Vet HC, Macaskill P, Irwig L (2013). Red flags to screen for malignancy in patients with low-back pain. Cochrane Database Syst Rev.

[34] Greenhalgh S, Selfe J (2009). A qualitative investigation of Red flags for serious spinal pathology. Physiotherapy.

[35] Green AJ, Jackson DA, Klaber Moffett JA (2008). An observational study of physiotherapists’ use of cognitive-behavioural principles in the management of patients with back pain and neck pain. Physiotherapy.

[36] Keogh A, Tully MA, Matthews J, Hurley DA (2015). A review of behaviour change theories and techniques used in group based self-management programmes for chronic low back pain and arthritis. Man Ther.

[37] Alexanders J, Anderson A, Henderson S (2015). Musculoskeletal physiotherapists’ use of psychological interventions: a systematic review of therapists’ perceptions and practice. Physiotherapy.

[38] Åsenlöf P, Denison E, Lindberg P (2005). Individually Tailored treatment targeting activity, motor behavior, and cognition reduces pain–related disability: a randomized controlled trial in patients with musculoskeletal pain. J Pain.

[39] Sanders T, Ong BN, Sowden G, Foster N (2014). Implementing change in physiotherapy: professions, contexts and interventions. J Health Organ Manag.

[40] Nicholas MK, George SZ (2011). Psychologically informed interventions for low back pain: an update for physical therapists. Phys Ther.

[41] Stevenson K, Lewis M, Hay E (2006). Does physiotherapy management of low back pain change as a result of an evidence-based educational programme?. J Eval Clin Pract.

[42] Bryant C, Lewis P, Bennell KL, Ahamed Y, Crough D, Jull GA (2014). Can physical therapists deliver a pain coping skills program? An examination of training processes and outcomes. Phys Ther.

[43] Joyce BR, Showers B (2002). Designing training and peer coaching: our needs for learning. Student achievement through staff development.

[44] Asan O, Montague E (2014). Using video-based observation research methods in primary care health encounters to evaluate complex interactions. Inform Prim Care.

[45] Mead N, Bower P (2000). Patient-centredness: a conceptual framework and review of the empirical literature. Soc Sci Med.

[46] Mead N, Bower P (2002). Patient-centred consultations and outcomes in primary care: a review of the literature. Patient Educ Couns.

[47] Hiller A, Guillemin M, Delany C (2015). Exploring healthcare communication models in private physiotherapy practice. Patient Educ Couns.

[48] Constand MK, MacDermid JC, Dal Bello-Haas V, Law M (2014). Scoping review of patient-centered care approaches in healthcare. BMC Health Serv Res.

[49] Parry R, Pino M, Faull C, Feathers L (2016). Acceptability and design of video-based research on healthcare communication: Evidence and recommendations. Patient Educ Couns.

[50] Ram G, Rethans S, Vleuten K (1999). Assessment of general practitioners by video observation of communicative and medical performance in daily practice: issues of validity, reliability and feasibility. Med Educ.

